# The Retention or Removal of the Sixth-Year Molar—Which?

**Published:** 1891-07

**Authors:** Theodore F. Chupein

**Affiliations:** Philadelphia.


THE
AMERICAN JOURNAL
—OF—
DENTAL SCIENCE.

VOL. XXV. BALTIMORE, JULY, 1891. NO. 3.

ARTICLE I.

### THE RETENTION OR REMOVAL OF THE SIXTH-YEAR MOLAR—WHICH?

BY THEODORE F. CHUPEIN, PHILADELPHIA.

[Read before the Odontological Society of Pennsylvania, January 3, 1891.

It is not the purpose to deny or affirm whether the sixth-year molar belongs to the temporary or the permanent denture, but it is the purpose to show that experience proves that its *timely* removal redounds to the benefit of the other teeth of the permanent set, and, in the large majority of instances, to the comfort of those we serve.

It has proved itself to our satisfaction that if this tooth be removed in time, the two bicuspids, on each side and in each jaw, will not be in such close contact as to endanger their proximate surfaces by decay, those surfaces which give us such care, solicitude, anxiety and trouble in our efforts to arrest caries when it has once gained a foothold. It has been demonstrated, in the large majority of cases, that removal at the proper age will induce the second or twelfth-year molar to work forward and occupy nearly the same place formerly occupied by the sixth-year molar, and this without tilting, leaning or assuming the least malposition, and that both the second molar, as well as (subsequently) the wisdom tooth, will take normal positions in the jaw with a free, easy space between each, whereby decay on the proximate surfaces of these molars as well as the bicuspids is largely the exception rather than the rule.

It will be admitted by nearly all, if not by all, dentists that decay on the proximate surfaces of the bicuspids and the three molars are the localities, *par excellence*, that are frequently most prone to decay, and are the surfaces most difficult to combat.

All statistics prove that the sixth-year molar is the tooth which decays more frequently, and on all surfaces. than any tooth of the denture, and that, like a decayed peach in a basket of such fruit, it communicates caries, by contact, to its neighbor (if caries is communicable in teeth, which we do not advance), and thus spreads ruin among the good seed.

The sixth-year molar, in my opinion, is often the bane of the denture, the black sheep of the flock. After the most careful work and the utmost solicitude on the part of both patient and operator, failure to preserve it is the result. Or, if it be preserved, is the victory commensurate with the labor or the pain incident to such victory. Or, are after-results beneficial to the rest of the teeth. I say no!

The jaws of the present generation do not seem to be large enough for the accommodation of thirty-two teeth. Witness the large number of cases of irregularity of the teeth which we see in people of our time, when the jaws have to be spread or enlarged to accommodate the teeth, or where teeth have to be extracted in order to make room. If, then, we can secure a better condition of the denture by the removal of this tooth, what good reason can be advanced for its retention?

It has been asserted that the second molar will tilt, and thus give a malocclusion with its antagonistic teeth, if the sixth year molar be extracted; but such a result will not occur if its removal be made at the proper age or time.

It has been advanced that the proper comminution or trituration of food cannot be accomplished as well if the sixth-year molar be removed as it can when it is retained. Yet we very much doubt this position, when, by its retention, frequently all the proximo-masticating surfaces of all the posterior teeth are so largely occupied by fillings that these fillings cannot offer as good means of communication as if the teeth were intact. Will it be contended that the proper trituration of food can be as well performed by teeth which are filled (however faithfully the contouring of such fi.lings restore the normal shape of the teeth) as it can be by genuine tooth-tissue? It has been asserted that if this tooth be extracted too soon it will cause an impairment of digestion; not only this, but, the bulk of mastication being thrown on the teeth anterior to it, this extraction will cause a change in the features of the patient, giving them an apish or bull dog expression. It may be doubted whether such might be the case if the extraction were done too soon, but nothing is suggested of this kind. At the time it is advised to extract this tooth, all the eight bicuspids will be fully developed, and the patient will not be without a molar to chew on for more than six or eight months at the farthest. Such a change in the features as is intimated, and such an impairment of the digestive functions, could not be brought about in so short a time with eight good back teeth to chew on. Indeed, very many adult persons go through life with sometimes even fewer posterior teeth, and these never think of resorting to artificial grinders to assist in mastication, being quite able to do all their chewing with eight bicuspids or less and no molars at all, and yet do not suffer from dyspepsia or have apish expression of countenance. It must not be concluded that it is advised to extract the sixth-year molar as soon as it gives pain from the effects of decay; such is not the idea. When this tooth shows early signs of decay, it would be better that every means known be used for its preservation up to a certain time of life, after which I would advise its extraction in the large majority of cases. The time for the proper extraction of the sixth-year molar, it must be stated, cannot be set down to any specified time any more than the erupting of the teeth can be positively determined, some of these erupting earlier and some later in different subjects; yet, ordinarily, from about the tenth and a half to the eleventh and a half year is near the period, the eleventh year most generally. I favor the extraction of these four teeth, but am inclined to the belief that the extraction of the lower should precede that of the upper molars by from six to nine months, because the lower teeth frequently erupt before the upper ones. A very popular dental writer has stated that "the overcrowded condition of the teeth causes caries, imperfect mastication, incorrect enunciation, and deformity of feature." But while I quote this author in support of the position, it is not to offer his testimony as binding, except in the matter of one—the "cause of caries"—because I do not believe he referred to the crowded condition of the teeth, except in so far as their malposition in the jaws was concerned. Yet I do hold that even in their regular position, when forced against each other in closer contact than is admissible from the size of the arch or intended by nature, such contact tends to, or is a most fruitful cause of, decay.

I do not wish to infer that decay is wholly due to close contact, for it is known that teeth which are widely separated are sometimes found with caries, and again, it may occur on surfaces that are never in contact, yet even when a tooth has begun to decay from contact with its neighbor, necessitating its removal, the decayed tooth seems, if not to assume a retrograde process, at least not to make as rapid progress in the consumption of toothtissue as it would have done had its neighbor been in place.

It will not be advanced that once a tooth has been filled it will not decay again. If such a consummation could be arrived at, it would be a joy indeed. It is the recurrence of caries after filling that makes the dentist seek the solution of the cause. This or that reason is advanced, most often the lack of thoroughness or faulty manipulation, yet the effect always remains. But whatever be its origin, the dentist must remedy the disaster, though he has to fill and refill again and again at each recurrence of decay.

When such proximo-masticating fillings increase in size at each recurrence, not only subjecting the patient to severe pain and strain in the preparation and filling, the dentist to great fatigue, the parent or relative to the payment of the fees these necessarily expensive operations entail, should not any means that promise the bringing about of different and improved results be adopted for humanity's sake? What is the use of continuing on a beaten track that does not lead quickly and most certainly to the coveted goal? And if the results, for the patient's benefit, can be brought about by the system advanced, is it not a duty we owe them to try it? And is it not often the case that the oft-repeated filling so demoralizes the patient that, in despair, rather than have the same thing done again, they resort to wholesale extraction, and hang their hopes, despite every argument to the contrary by their dental adviser, in the substitution of artificial teeth?

But it may be advanced, "If such a condition could be brought about, what would become of the dentist's art? It is caries which dentistry has to combat, and principally in those teeth and those localities which furnish the bulk of employment in dentistry." I am sure that it is only those who are actuated by cupidity or mercenary motives who would bring forward an argument like this in rebuttal. Dentistry desires to be allied with medicine and surgery; but can an instance be shown where a physician has ever failed to advocate a measure that would point to or foster a system for the prevention of disease? May it not have been said that prevention of disease would bring about the downfall of medicine? Yet, did that prevent the noble Jenner and the equally noble Koch from promulgating their systems for the promotion or cure of small-pox and consumption? These diseases are wasting and tedious, and would naturally furnish a very large employment to the physician; but can human suffering and personal aggrandizement ever be placed in the balance?

I assure you I am in earnest in this matter, and have my patients' interests as much at heart as any man among you, and I feel sure that its performance will not only save the patient very many severe and painful operations, but the dentist severe and tiresome work.

The evidences of the truth of this position are to be seen in operations performed, of the like character, by other dentists, and in all cases seen benefit has resulted.

I present you an ideal case in the model sent around. You will perceive on one side of the mouth, where the sixth-year molar has been retained, how all the proximo-masticating surfaces of the teeth have been filled extensively; whereas, on the other side, where that tooth has been removed, how all the proximate surfaces have escaped decay. This is attributed to the freedom from close contact and the facility with which these surfaces can be kept clean with little trouble to the patient. It may be considered good theory from æsthetic grounds to retain the sixth-year molar, but I know it is good practice for our patient's comfort to remove it.

Is it not more reconcilable with common sense to save twenty-eight out of the thirty-two teeth which are given to man than to make the attempt to save the whole thirty-two and fail in the attempt? For is it not a failure, or next door to it, when (whatever may be the cause of the recurrence of decay) cavities, once filled, have to be refilled again and again in order to keep back the inroads of the insidious enemy?

Is it not more reasonable, and will it not be admitted, that the patient's interest, health and comfort are better served, to say nothing of the operator's, when twenty-eight teeth are preserved, with only, perhaps, a few simple places filled, than to try to save the thirty-two, with such results as are observed in the large majority of cases? And would not any patient prefer to have this many teeth, almost, if not quite, intact, rather than thirty-two tumble-down crowns half tooth and half gold, if the case were submitted to them? In a remarkably fine paper recently read before the Southern Dental Association by Dr. W. H. Atkinson, he uses the following language: "The highest use of any profession is to so instruct the people that they shall be in such healthful condition as not to demand a remedy. There is no reason why dentists should not be entirely competent to prevent disease more often than to treat it. The time has come when prevention is more important for them to turn their attention to than treatment." Who will not admit that he is a better physician who can prevent disease than he who can cure it? So that the old proverb of "an ounce of prevention is better than a pound of cure" is an acknowledged truth.

We could advance many arguments in further proof of our position, but we have said, perhaps, more than enough to set the matter before you, and therefore leave it for your consideration.—*International Dental Journal.*

